# A Survey of Robotics Control Based on Learning-Inspired Spiking Neural Networks

**DOI:** 10.3389/fnbot.2018.00035

**Published:** 2018-07-06

**Authors:** Zhenshan Bing, Claus Meschede, Florian Röhrbein, Kai Huang, Alois C. Knoll

**Affiliations:** ^1^Chair of Robotics, Artificial Intelligence and Real-time Systems, Department of Informatics, Technical University of Munich, Munich, Germany; ^2^Department of Data and Computer Science, Sun Yat-Sen University, Guangzhou, China

**Keywords:** spiking neural network, brain-inspired robotics, neurorobotics, learning control, survey

## Abstract

Biological intelligence processes information using impulses or spikes, which makes those living creatures able to perceive and act in the real world exceptionally well and outperform state-of-the-art robots in almost every aspect of life. To make up the deficit, emerging hardware technologies and software knowledge in the fields of neuroscience, electronics, and computer science have made it possible to design biologically realistic robots controlled by spiking neural networks (SNNs), inspired by the mechanism of brains. However, a comprehensive review on controlling robots based on SNNs is still missing. In this paper, we survey the developments of the past decade in the field of spiking neural networks for control tasks, with particular focus on the fast emerging robotics-related applications. We first highlight the primary impetuses of SNN-based robotics tasks in terms of speed, energy efficiency, and computation capabilities. We then classify those SNN-based robotic applications according to different learning rules and explicate those learning rules with their corresponding robotic applications. We also briefly present some existing platforms that offer an interaction between SNNs and robotics simulations for exploration and exploitation. Finally, we conclude our survey with a forecast of future challenges and some associated potential research topics in terms of controlling robots based on SNNs.

## 1. Introduction

The mysterious biological intelligence of living creatures has long attracted us to explore their capabilities from perceiving, memorizing, to thinking, and then resulting in languages and behaviors. Nowadays, owing to the increasing efforts of mimicking those structural and functional principles, scientists have investigated how the brain, robot actuators, and sensors could work together to operate robots autonomously performing complex tasks, e.g., in the form of self-driving vehicles (Schoettle and Sivak, 2014), biomimetic robots (Ijspeert et al., 2007; Gong et al., 2016), collaborative industrial robots (Shen and Norrie, 1999). However, to acquire more autonomy and operate within the real world, robots should be further investigated with the following capacities: (1) perceiving their environments via sensors that typically deliver high-dimensional data; (2) processing redundant or sparse information with low response latency and energy efficiency; (3) behaving under dynamic and changing conditions, which requires a self-learning ability.

Meanwhile, neither traditional control strategies nor conventional artificial neural networks (ANNs) can meet those aforementioned needs. To be specific, traditional model-based control methods via numerical techniques, kinematics and dynamics approaches often fail to adapt to unknown situations (Ijspeert, 2008; Yu et al., 2014; Bing et al., 2017). On the other hand, conventional ANNs have difficulties in processing the high computational demands for a step further, despite the hardware progress that made large neural networks applicable to real-world problems. The main disadvantages are as follow. First, training artificial neural networks is time consuming (Krogh and Vedelsby, 1995) and can easily take multiple days for state-of-the-art architectures (Lee C. S. et al., 2016). Training large-scale networks is computationally expensive [*AlphaGo* 1,202 CPUs and 176 GPUs (Silver et al., 2016)], and running them typically produces high response latencies (Dong et al., 2009). Second, performing computations with large networks on traditional hardware usually consumes a lot of energy as well. In self-driving cars for example, this results in computational hardware configurations that consume a few thousand Watts compared to the human brain, which only needs around 20 Watts of Power (Drubach, 2000). Especially in mobile applications, these are considerable disadvantages, in which real-time responses are important and energy supply is limited.

In nature, information is processed using relatively small populations of spikes and their precise relative timing, which is sufficient to drive learning and behavior (VanRullen et al., 2005; Houweling and Brecht, 2008; Huber et al., 2008; Wolfe et al., 2010). Therefore, a promising solution to robotics control challenges could be given by spiking neural networks that mimic the underlying mechanisms of the brain much more realistically. Due to their functional similarity to the brain, SNNs have the capabilities for processing information and learning in a much better fashion, both in terms of energy and data, e.g., building large-scale brain model (Eliasmith et al., 2012) or using neurally inspired hardware such as the SpiNNaker board (Furber et al., 2014) or Dynamic Vision Sensors (DVS) (Lichtsteiner et al., 2008). Moreover, SNNs have offered solutions to a broad range of specific implementations, such as fast signal-processing (Rossello et al., 2014), speech recognition (Loiselle et al., 2005), robot navigation (Nichols et al., 2010), and other problems solved by non-spiking neural networks, but in fact showed even more superiorities. However, a comprehensive review on controlling robots based on spiking neural networks is still missing.

Therefore, in this article, we aim to survey the state-of-the-art SNN modeling, design, and training methods for controlling a variety of robotics applications since the recent decade. The overall motivation of this article is to ease the barrier for roboticists to understand the complicated biological knowledge of SNNs, meanwhile enlighten readers with some general learning-based SNN approaches to different robot control tasks. The contribution of this survey is three-fold. First, we try to set forth SNNs' superiorities in terms of speed, energy efficiency, and computation capabilities. And we outline a general design framework for controlling SNN-based robotics tasks. Then, our survey aims to summarize the learning-based SNNs in robotics tasks, ranging from the modeling, learning rules, and robot implementations and platforms. We generally categorize the selected robotic implementations according to different learning rules, this shows up in the source of learning signals, which could be acquired from different ways, such as the labeled dataset, neutral stimulus, rewards from environment or other external controllers. Finally, we attempt to point out the open topics that need to be addressed for implementing SNNs in robotics tasks.

The rest of the article is organized as follows. In section 2, the theoretical background of SNNs will be briefly introduced, which will include their biological foundations and the changing course of the artificial neural networks on a basis of learning rules. Section 3 presents the primary motivation and research framework for SNN-based robot control. Then we will discuss SNN implementations from the simple neuron unit to the topologies of more advanced systems (section 4). Various methods training SNNs for control tasks will be classified and explained with their corresponding robotic applications (section 5), as well as the existing platforms for exploring neurorobotics (section 6). Finally we will summarize future challenges and potential research topics in section 7 and conclude in section 8.

## 2. Theoretical background

Before studying in deep of the robotics control based on SNNs, it is worth briefly summarizing the biological mechanisms taking place in human nervous system. Therefore, this section serves as a short summary of the theoretical foundations as well as the vocabulary that is used in the following sections. An in-depth introduction of SNNs can be found in Vreeken (2003), Ghosh-Dastidar and Adeli (2009), Ponulak and Kasinski (2011), and Grüning and Bohte (2014).

### 2.1. Biological background

Even today's understanding of the human brain remains rather incomplete and challenging, some insights into our neural structure have been made over the past couple of decades. Since the initial discovery of neurons as the basic structure of the nervous system by Santiago Ramón y Cajal at the beginning of the twentieth century, a rough concept of how neurons might work has been developed. At the very basis, neurons can be understood as simple building blocks processing incoming information in the form of short pulses of electrical energy into output signals. By connecting neurons to huge networks, complex dynamics emerge that can process information and make sense of our world. This basic concept can be found all over nature, ranging from simpler organisms like jellyfish with a couple of thousand neurons to humans with an estimated number of 86 billion neurons on average in our nervous system (Herculano-Houzel, 2012).

**Figure 1:**
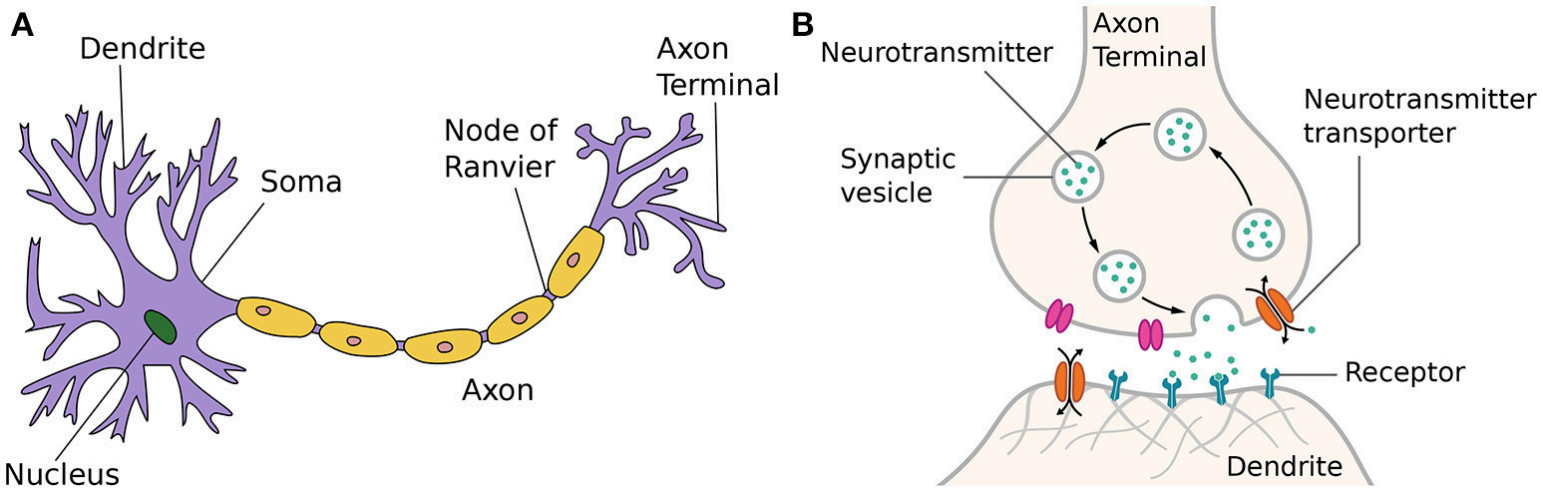
**(A)** Neuron (Wikipedia, 2017b). The figure is attributed to Quasar Jarosz at English Wikipedia, CC BY-SA 3.0, https://commons.wikimedia.org/w/index.php?curid=7616130. **(B)** Synapse (Wikipedia, 2017e). The figure is attributed to Thomas Splettstoesser (https://www.scistyle.com) - Own work, CC BY-SA 4.0, https://commons.wikimedia.org/w/index.php?curid=41349545.

The structure of a typical neuron of the human brain embedded in a salty extra-cellular fluid is shown in Figure 1A. Incoming signals from multiple dendrites alter the voltage of the neuronal membrane. When a threshold is reached, the cell body or soma sends out an action potential—also called spike or pulse—itself. This process of generating a short (1ms) and sudden increase in voltage is usually referred to as spiking or firing of a neuron. After firing, it is followed by a short inactive period called the refractory period, in which the neuron cannot send out other spikes regardless of any incoming signals.

Once the membrane potential threshold has been reached and the neuron fires, the generated output spike is transmitted via the axon of a neuron. These can grow quite long and branch out to a multitude of other nervous cells at the end.

At the end of an axon, the axon terminal, incoming signals are transmitted to other nervous cells, such as other neurons or muscular cells. There is now proven evidence that synapses are in fact one of the most complicated part of a neuron. On top of transmitting information, they work as a signal pre-processor and play a crucial part in learning and adaption for many neuroscientific models. When a traversing spike reaches an axon terminal, it can cause a synaptic vesicle to migrate toward the presynaptic membrane, as shown in Figure 1B. At the presynaptic membrane, the triggered vesicle will fuse with the membrane and release its stored neurotransmitters into the synaptic cleft filled with the extra-cellular fluid. After diffusing into this gap, neurotransmitter molecules have to reach a matching receptor at the postsynaptic side of the gap and bind with them. Directly or indirectly, this causes postsynaptic ion-channels to open or close. The resulting ion flux initiates a cascade that traverses the dendritic tree down to the trigger zone of the soma, changing the membrane potential of the postsynaptic cell. Therefore, different neurotransmitters can have opposing effects on the excitability of postsynaptic neurons, thus mediating the information transfer. These effects that make postsynaptic cells either more or less likely to fire action potentials are called excitatory postsynaptic potential or inhibitory postsynaptic potential, respectively. The dependence of postsynaptic potentials on different amounts and types of neurotransmitters released and the resulting number of ion-channels activated is often referred to as synaptic efficacy in short. After a while, neurotransmitter molecules are released again from their receptors into the synaptic cleft and either reabsorbed into the presynaptic axon terminal or decomposed by enzymes in the extra-cellular fluid.

The properties, characteristics, and capacities of synapses as signal pre-processors, e.g., chances of vesicle deployment or regeneration and amount of receptors, are not fixed, but always changing depending on the short and long-term history of its own and outside influences. Neuro-hormones in the extra-cellular fluid can influence both the pre and postsynaptic terminals temporarily, i.e., by enhancing vesicle regeneration or blocking neurotransmitters from activating ion-gate receptors. All these effects that change the influence of incoming spikes on the postsynaptic membrane potential are usually referred to as synaptic plasticity and form the basis of most models of learning in neuro and computer-sciences.

### 2.2. From mcculloch-pitts to backpropagation

In 1943, neurophysiologist Warren McCulloch and mathematician Walter Pitts wrote a theoretical paper on how neurons might work describing a simple neural network model using electrical circuits (McCulloch and Pitts, 1943). Capable of performing mathematical operations with boolean outputs, these first generation neural networks fire binary signals once a threshold of summed incoming signals is reached in a neuron. They have been successfully applied in powerful artificial neural networks such as multi-layer perceptrons and Hopfield nets (Hopfield, 1982).

With the advent of more powerful computing, this concept was later extended by introducing continuous activation functions, e.g., sigmoid (Han and Moraga, 1995) or hyperbolic tangent functions, to handle analog inputs and outputs as well. In fact, it has been proven that sufficiently large neural networks with continuous activation functions can approximate any analog function arbitrarily well by altering the network information flow through their synaptic weights (Hornik et al., 1989). The most commonly used and powerful supervised learning algorithm that takes advantage of continuous activation functions by using gradient-descent on an error function is called backpropagation (Hecht-Nielsen, 1992).

However, second generation neurons do not model electrical pulses that have been described in their biological counterparts, their analog information signals can actually be interpreted as an abstracted rate coding. Over a certain period of time, an averaging window mechanism can be used to code pulse frequencies into analog signals giving these models a more biologically plausible meaning.

### 2.3. Spiking neural networks

Following its biological counterpart, a third generation of neural networks (Maass, 1997, 2001) has been introduced that directly communicates by individual sequences of spikes. Instead of using abstracted information signals, they use pulse-coding mechanisms that allow for the incorporation of spatial-temporal information that would otherwise be lost by averaging over pulse frequencies. It becomes clear that these neural network models, referred to as Spiking-Neural-Networks (SNNs), can be understood as an extension to second generation neural networks and can, in fact, be applied to all problems solvable by non-spiking neural networks (Fiasché and Taisch, 2015). In theory, it has been shown that these models are even more computationally powerful than perceptrons and sigmoidal gates (Maass, 1997).

Due to their functional similarity to biological neurons (DasGupta and Schnitger, 1992), SNNs have become a scientific tool for analyzing brain processes, e.g., helping to explain how the human brain can process visual information in an incredibly short amount of time (Chun and Potter, 1995). Moreover, SNNs promise solutions for problems in applied engineering as well as power efficient, low-latency alternatives to second generation neural networks, e.g., for applications in robotics (Lee J. H. et al., 2016).

## 3. Primary motivation and framework

In this section, we will briefly introduce the research impetuses of SNN-based robotics control from multiple aspects. Core points and a major framework for generally organizing an SNN for robotic implementation are introduced as well.

### 3.1. Primary impetuses

As the third generation of the neural network model, SNNs have attracted more and more attention and gradually become an interdisciplinary research field for neuroscience as well as robotics. For clarity and simplicity, the fascinating features of SNNs, which apply well to robotic controllers, can be summarized as follows.

#### 3.1.1. Biological plausibility

From the perspective of neuroscience, SNNs once again raise the level of biological realism by directly using individual sequences of spikes in communication and computation, like real neurons do (Ferster and Spruston, 1995). Experimental evidence accumulated during the last few years has indicated that many biological neural systems use the timing of single-action potentials (or “spikes”) to encode information (Maass, 1997), rather than the traditional rate-based models. In Walter et al. (2015a), it is explained that how the exact modeling of time in spiking neural networks serves as an important basis for powerful computation based on neurobiological principles.

#### 3.1.2. Speed and energy efficiency

Despite the hardware upgrades that make large neural networks applicable to real-world problems, it usually does not apply to robotics platforms with limited energy and computing resources. Since SNNs are able to transmit and receive large volumes of data encoded by the relative timing of only a few spikes, this leads to the possibility of very fast and efficient implementations. For example, experiments have demonstrated that visual pattern analysis and pattern classification can be carried out by humans in just 100 ms, in spite of the fact that it involves a minimum of 10 synaptic stages from the retina to the temporal lobe (Thorpe et al., 2001). On the other hand, in terms of energy efficiency, maintaining sufficient working of the nervous system to perform various tasks requires a continuous energy supply (Sengupta and Stemmler, 2014). Yet, the human brain only needs very low power consumption, which is around 20 W of Power (Drubach, 2000).

#### 3.1.3. Computational capabilities

Recently, established experiments *in vivo* have indicated that SNNs are capable of processing the information sufficiently using a relatively small number of spikes to drive learning and behavior (VanRullen et al., 2005; Houweling and Brecht, 2008; Huber et al., 2008; Wolfe et al., 2010); meanwhile, they can also handle a large-scale network containing up to a trillion neurons like elephants (Herculano-Houzel et al., 2014). Furthermore, SNNs are superior to non-spiking ones for utilizing the temporal information, referring to the precise timing of events to acquire the exact information with incredible precision and accuracy. For instance, the auditory system of the barn owl is able to locate sources of sound in the horizontal plane with a precision of 1 to 2 degrees, which equates to a temporal difference of only a few microseconds (5μ*s*) between the arrival of sound waves at the left and right ears (Gerstner et al., 1999).

#### 3.1.4. Information processing

Instead of using abstracted information signals, SNNs use pulse coding mechanisms that allow incorporating spatial-temporal information that would otherwise be lost by only averaging over pulse frequencies. This ability to learn and act in a dynamic environment, rich with temporal information, is a necessary quality for biological systems and for artificial systems that seek to perform similar tasks. Neurobiologists used weekly electric fish as a model to study the processing from stimulus encoding to feature extraction (Gabbiani et al., 1996; Metzner et al., 1998). They found that although pyramidal cells do not accurately convey detailed information about the time course of the stimulus, they reliably encode up- and down-strokes of random modulations by bursts of spikes. In addition, a problem referred to as “dynamic binding,” has at best remained elusive to implement in neural networks, referring to different types of sensor information together in an assembly. SNNs are able to efficiently detect conjunctions of primitives (features) anywhere on a-large-input grid in an efficient, position-invariant manner. Examples such as data classification and image recognition tasks can be found in Hopfield (1995), Thorpe et al. (2001), Bohte et al. (2002), Guyonneau et al. (2004), and Shin et al. (2010).

**Figure 2:**
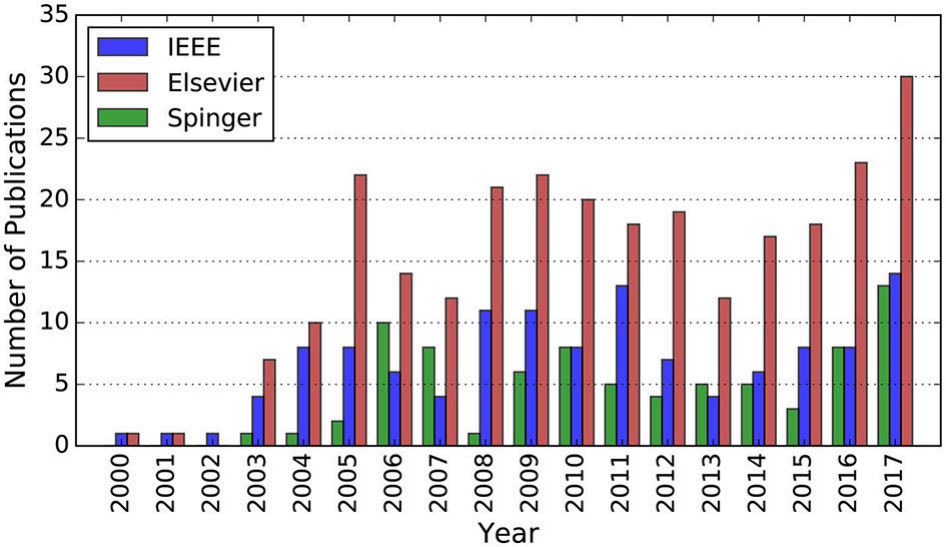
Number of publications whose abstract contains the terms “robot” and “spiking neural network” in the IEEE Explore and Elsevier Scopus database, respectively. Number of publications whose title contains the terms “robot” and its main text contains the term “spiking neural network” in the Springer database. All the data is from 2000 to 2016.

In conclusion, these interesting features make SNNs suitable for pursuing autonomy for robotics implementations. However, there is an implicit knowledge gap since SNNs are just investigated at the theoretical level, rather than widely adopted to practical robotics applications. Even so, the growing knowledge of spiking neural networks and their increasingly popularity consistently draw more research attention and have led to more and more SNN-based implementations, as illustrated in Figure 2.

### 3.2. Research directions

**Figure 3:**
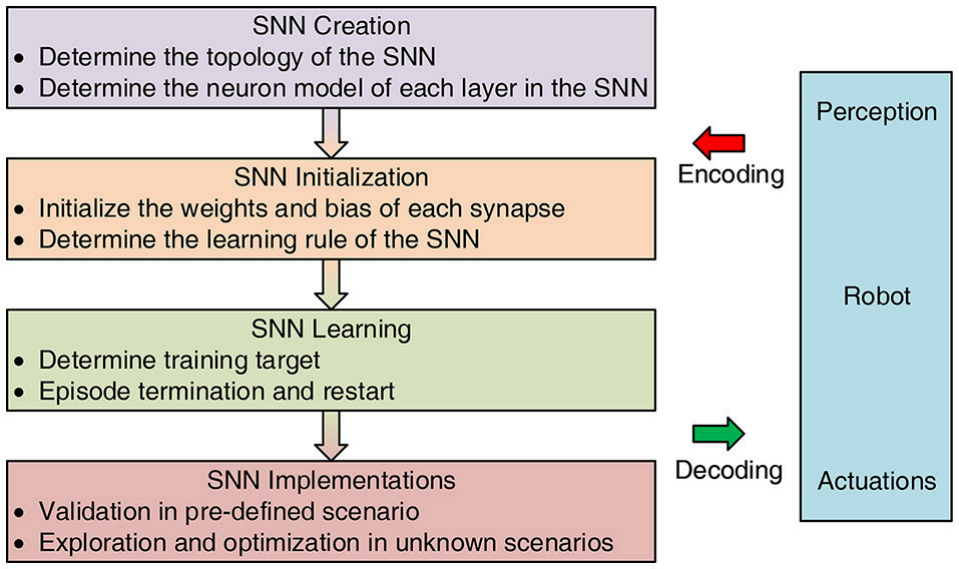
General design framework for learning-inspired SNN-based robot control.

The major research directions of SNNs have focused on three aspects: SNN modeling, training, and implementations, which will be detailedly discussed or briefly introduced with other reference readings in the following sections. A general design framework for learning-inspired robot control is shown in Figure 3. Most robot control tasks chasing autonomy could be described as a cycle including three steps, namely, perception, decision, execution (Gspandl et al., 2012). Robots usually use their sensors and actuators to sense and interact with the environment. However, the SNNs can be regarded as the brains to make decision, which build up a bridge between perception and execution, by taking encoded information from environment and outputting decoded motor commands for robots. To be specific, first, the architecture and mathematical model of an SNN should be determined including the neuron and synapse. Neurons are known to be a major signaling unit of the nervous system, and synapses can be seen as signal transmitters that communicate among neurons. For this reason, modeling of an SNN is of great importance to characterize its properties. Then, the SNN should be initialized and trained with specific parameters and learning rules, as conventional neural networks. Choosing an appropriate learning rule directly impact the performance of the networks. For an SNN, the most common learning rule is the Hebbian rule, which will be explained in the following section. Finally, after training the SNN successfully, it should be validated in other scenarios and be optimized if necessary.

## 4. Modeling of spiking neural networks

At the very beginning of the construction of an SNN for robot control, an appropriate SNN control model should be decided on. The basic task is to determine the general topological structure of the SNN, as well as the neuron models in each layer of the SNN.

### 4.1. Neuron models

Generally, neuron models can be expressed in the form of ordinary differential equations. In the literature, many different mathematical descriptions of spiking neural models have been proposed, processing excitatory and inhibitory inputs using internal state variables. The most influential models used for SNNs are the Hodgkin-Huxley model (Hodgkin and Huxley, 1952) as well as the Integrate-and-Fire model and its variants (Burkitt, 2006). To find an appropriate one among existing diverse neuron models, there is usually a trade-off to be balanced between the biological plausibility and complexity. A detailed comparison of the neuro-computational properties of spiking and bursting models can be found in Izhikevich (2004).

One of the most widely used models is the so-called Leaky-Integrate-and-Fire (LIF) model (Stein, 1965) that can be easily explained by the principles of electronics. These models are based on the assumption that the timing of spikes, rather than the specific shape, carries neural information (Andrew, 2003). The sequences of firing times are called spike trains and can be described as

(1) $$\begin{array}{r} S\left(t\right)=\underset{f}{\sum }\delta \left(t-t^{f}\right), \end{array}$$

where *f* = 1, 2, … is the label of a spike and δ(·) is a Dirac function defined as

(2) $$\begin{array}{r} \delta \left(x\right)=\left\{ \begin{array}{cc} +\infty \; & \text{if}x=0\\ 0\; & \text{if}\;x\neq 0 \end{array}\right. \end{array}$$

(3) $$\begin{array}{r} \int_{-\infty }^{\infty }\delta \left(t\right)dt=1. \end{array}$$

Passing a simplified synapse model, the incoming spike train will trigger a synaptic electric current into the postsynaptic neuron. This input signal *i*(*t*) induced by a presynaptic spike train *S*_*j*_(*t*) can, in a simple form, be described by the exponential function (Ponulak and Kasinski, 2011):

(4) $$\begin{array}{r} i\left(t\right)=\int_{0}^{\infty }S_{j}\left(s-t\right)\exp\left(-s/\tau_{s}\right)ds. \end{array}$$

Here, τ_*s*_ denotes the synaptic time constant. This synaptic transmission can be modeled by low-pass filter dynamics.

The postsynaptic current then charges the LIF neuron model increasing the membrane potential *u* according to

(5) $$\begin{array}{r} \tau_{m}\frac{du}{dt}\left(t\right)=u_{rest}-u\left(t\right)+R\left(i_{0}\left(t\right)+\sum w_{j}i_{j}\left(t\right)\right). \end{array}$$

where τ_*m*_ = *RC* is the time constant of the neuron membrane, modeling the voltage leakage, depending on the resistance *R*. *u*_*rest*_ is the potential value after each reset. *i*_0_(*t*) denotes an external current driving the neural state, *i*_*j*_(*t*) is the input current from the *j*_*th*_ synaptic input and *w*_*j*_ represents the strength of the *j*_*th*_ synapse. Once the membrane potential *u* reaches a certain firing threshold ϑ, the neuron fires a single spike and its membrane potential is set back to *u*_*rest*_. Usually, this spiking event is followed by a refractory period in which the neuron stays inactive and can't be charged again.

It is worth pointing out that biological studies highlight the presence of another operational unit *cell assemblies* (Braitenberg, 1978) in the brain, which are defined as a group of neurons with strong mutual excitatory connections and tend to be activated as a whole. A deeper review of spiking neuron models can be found in Andrew (2003).

### 4.2. Information Encoding and Decoding

The term neural encoding refers to representing information from the physical world (such as direction of a moving stimulus) in the activity of a neuron (such as its firing rate). On the other hand, information decoding is a reverse process to interpret from neuron activity to electrical signal for actuators (such as muscle or motor). How the brain encodes information is to think of two spaces: the physical space and neural space. The physical space can be the physical properties of objects, such as color, speed, and temperature. Neural space consists of properties of a neuron, such as firing rate in most cases.

A number of neural information encoding methods have been proposed, such as binary coding (Gütig and Sompolinsky, 2006), population coding (Probst et al., 2012), temporal coding, and the most commonly used rate coding (Urbanczik and Senn, 2009; Wade et al., 2010). For binary coding, neurons are only modeled to take two values *on/off* , but it ignores the timed nature and multiplicity of spikes altogether. Due to is simplicity, this coding mechanism was used in early-stage implementations. Besides, binary coding is also used to represent pixel value of an image (Meschede, 2017). For rate coding, it is inspired by the observation that neurons tend to fire more often for stronger (sensory or artificial) stimulus. Scientists usually use a concept in probability theory known as the Poisson process to simulate spike trains that have characteristics close to real neurons. As the most intuitive and simple coding strategy, rate-coding has been adopted by most robotic implementations. For temporal coding, it is motivated by the evidence founded in neuroscience that spike-timing can be remarkably precise and reproducible Gerstner et al. (1996). With this encoding strategy, information is represented with the timing when the spike occurs. However, the underlying mechanism is still not so clear. The aforementioned coding solutions is usually for one single neuron. However, sometime a population of neurons is used as a whole to encode information. This is strongly supported by the brain of living creature, where functions are controlled by one area of neuron populations.

The goal of neural decoding is to characterize how the electrical activity of neurons elicit activity and responses in the brain. The most common used scheme for decoding is rate-based, where stronger neuron activity usually means higher motor speed or force. In Kaiser et al. (2016), a steering wheel model based on an agonist-antagonist muscle system was proposed according to the spike numbers of output neuron.

### 4.3. Synaptic plasticity models

Once the neuron model is decided on, the synapse model should be carefully chosen to connect those neurons inside and among the layers of SNNs. By influencing the membrane potentials of each connected neuron, synaptic plasticity was first proposed as a mechanism for learning and memory on the basis of theoretical analysis (Hebb, 1949). Up to this day, the synaptic plasticity models used for practical implementations are typically very simple. Based on an input-output relationship between neuronal activity and synaptic plasticity, they are roughly classified into two types, which are rate-based and spike based, that differ in the type of their input variables.

#### 4.3.1. Rate-based

The first and most commonly used definition of a firing rate refers to a spike-count average over time (Andrew, 2003). The rate-based model is a popular approach for converting conventional ANNs into a spiking neural network that can still be trained by backpropagation. It has been successfully used in many aspects, especially in experiments on the sensory or motor system (Adrian, 1926; Bishop, 1995; Kubat, 1999; Kandel et al., 2000).

#### 4.3.2. Spike-based

Spike-based learning rules were developed in Gerstner et al. (1993), Ruf and Schmitt (1997), Senn et al. (1997), Kempter et al. (1999), and Roberts (1999). Experiments showed that the synaptic plasticity is influenced by the exact timing of individual spikes, in particular, by their order (Markram et al., 1997; Bi and Poo, 1998). If a presynaptic spike preceded a postsynaptic spike, a potentiation of the synaptic strength could be observed, while the reversed order caused a depression. This phenomenon has been termed as Spike-Timing-Dependent-Plasticity (STDP) or anti-STDP for the exact opposite impact and explains the activity-dependent development of nervous systems. In other words, neural inputs that are likely to have contributed to the neurons' excitation are strengthened, while inputs that are less likely to have contributed are weakened. As for neuro-engineering, STDP has demonstrated to be successfully implemented as the underlying neural learning mechanism in robots and other autonomous systems in both simulated and real environments.

In the past, different mathematical models of STDP have been proposed, e.g., by Gerstner and Kistler (2002). For this work, the weight update rule under STDP as a function of the time difference between pre and postsynaptic spikes was defined as

(6) $$\begin{array}{rc} \Delta t & =t_{post}-t_{pre} \end{array}$$

(7) $$\begin{array}{rc} STDP\left(\Delta t\right) & =\left\{ \begin{array}{cc} A_{+}e^{-\Delta t/\tau_{+}},\; & \text{if}\Delta t\geq 0\\ -A_{-}e^{\Delta t/\tau_{-}},\; & \text{if}\Delta t<0 \end{array}\right., \end{array}$$

with *A*_+_ and *A*_−_ representing positive constants scaling the strength of potentiation and depression, respectively. τ_+_ and τ_−_ are positive time constants defining the width of the positive and negative learning window. For deeper insights into the influence of the STDP mechanism, readers could refer to Song et al. (2000), Rubin et al. (2001), and Câteau and Fukai (2003).

A comparison of rate-based and spike-based spiking neural networks used for MNIST classification is shown in Diehl and Cook (2015).

### 4.4. Network models

The SNN network model resembles the synapse model in that it simulates synaptic interactions among neurons. Typical examples of neural networks consisting of neurons of these types are classified into two general categories:

#### 4.4.1. Feed-forward networks

As the first and simplest type of network topology, information in feed-forward networks always travels from the input nodes, through hidden nodes (if any), to the output nodes and never goes backwards. In the biological nervous system, abstracted feed-forward networks are mainly found to acquire and transmit external information. Therefore, similarly, networks of this type are usually adopted for low-level sensory acquisition in robotic systems, such as vision (Perrinet et al., 2004), tactile sensing (Rochel et al., 2002), and olfaction (Cassidy and Ekanayake, 2006). For example, inspired by the structures and principles of primate visual cortex, Qiao et al. (2014, 2015, 2016) enhanced the feed-forward models including Hierarchical Max Pooling (HAMX) model and Convolutional Deep Belief Network (CDBN) with memory, association, active adjustment, semantic and episodic feature learning ability etc., and achieved good results in visual recognition task.

**Figure 4:**
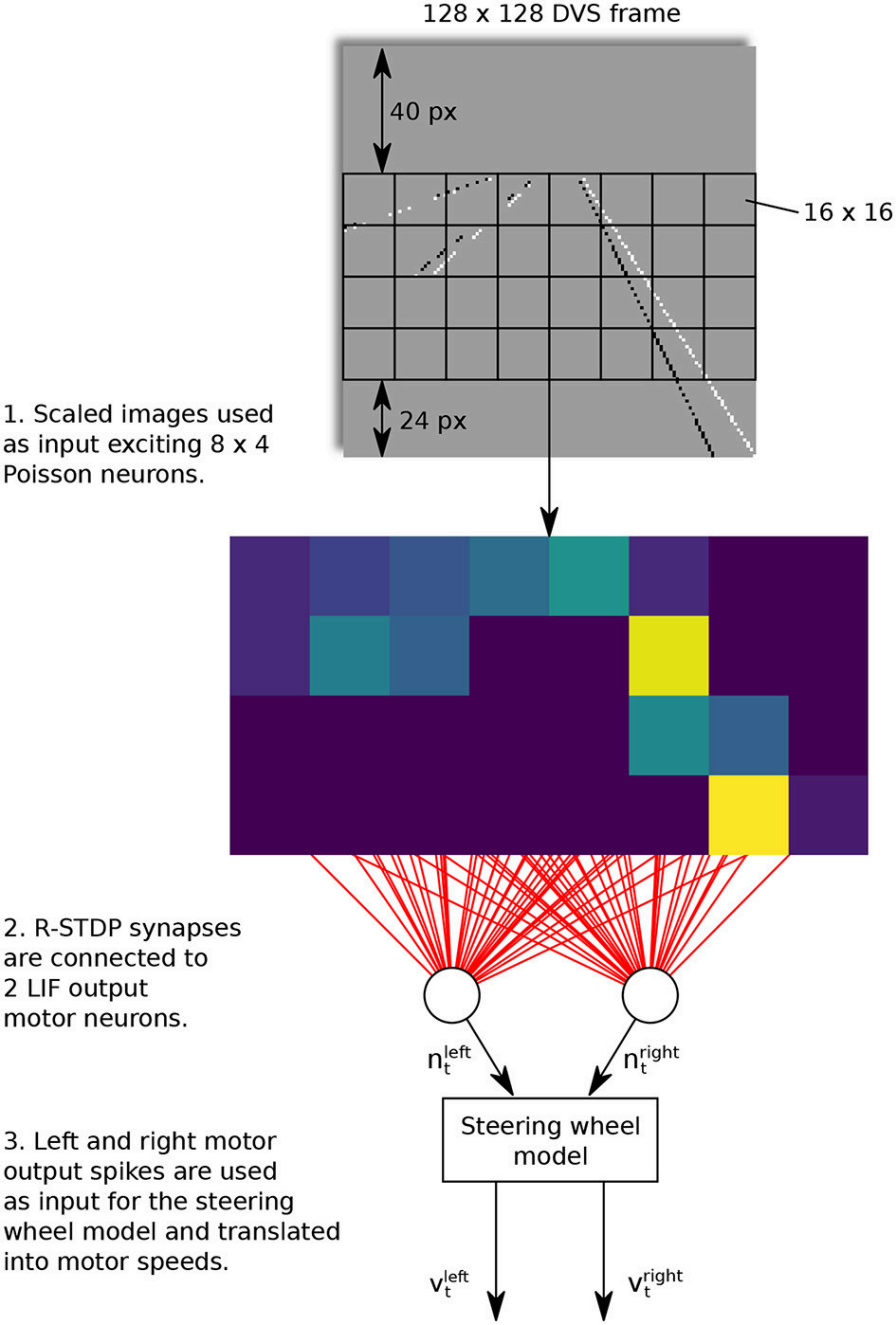
Control architecture of feed-forward SNN. A R-STDP SNN is used to achieve lane-keeping task. The sensor input is the event sequence from DVS and the two LIF output neurons are used to decode motor speed. All these neurons are connected with R-STDP synapse in an “all to all” fashion.

Taking the work from Meschede (2017) as an example, a two-layer feed-forward SNN was trained for a lane keeping vehicle. The control scheme is shown in Figure 4. In this work, the dynamic vision sensors (DVS) was used to detect the land markers by generating a sequence of events. The input layer consisted of 8 × 4 Poisson neurons and connected to the two LIF output motor neurons with R-STDP synapses in an “all to all” fashion. The learning phase was conducted by repeatedly training and switching the robot from the start positions in the inner and outer lanes. In comparison with other three learning methods, namely, the deep Q-learning (DQN), DQN-SNN, and Braitenberg Vehicle, the R-STDP SNN exhibited the best accuracy and adaptability in different lane scenarios.

#### 4.4.2. Recurrent networks

Different from the feed-forward networks, recurrent neural networks (RNNs) transmit their information with a directed cycle and exhibit dynamic temporal behaviors. It is worth pointing out that recurrent neural networks are recursive neural networks (Wikipedia, 2017d) with a certain structure such as a linear chain. Living organisms seem to use this mechanism to process arbitrary sequences of inputs with their internal memory stored inside RNNs. As for robotics implementations, RNNs are widely used for vision (Kubota and Nishida, 2006), planning (Soula et al., 2005; Rueckert et al., 2016), and dynamic control (Probst et al., 2012).

**Figure 5:**
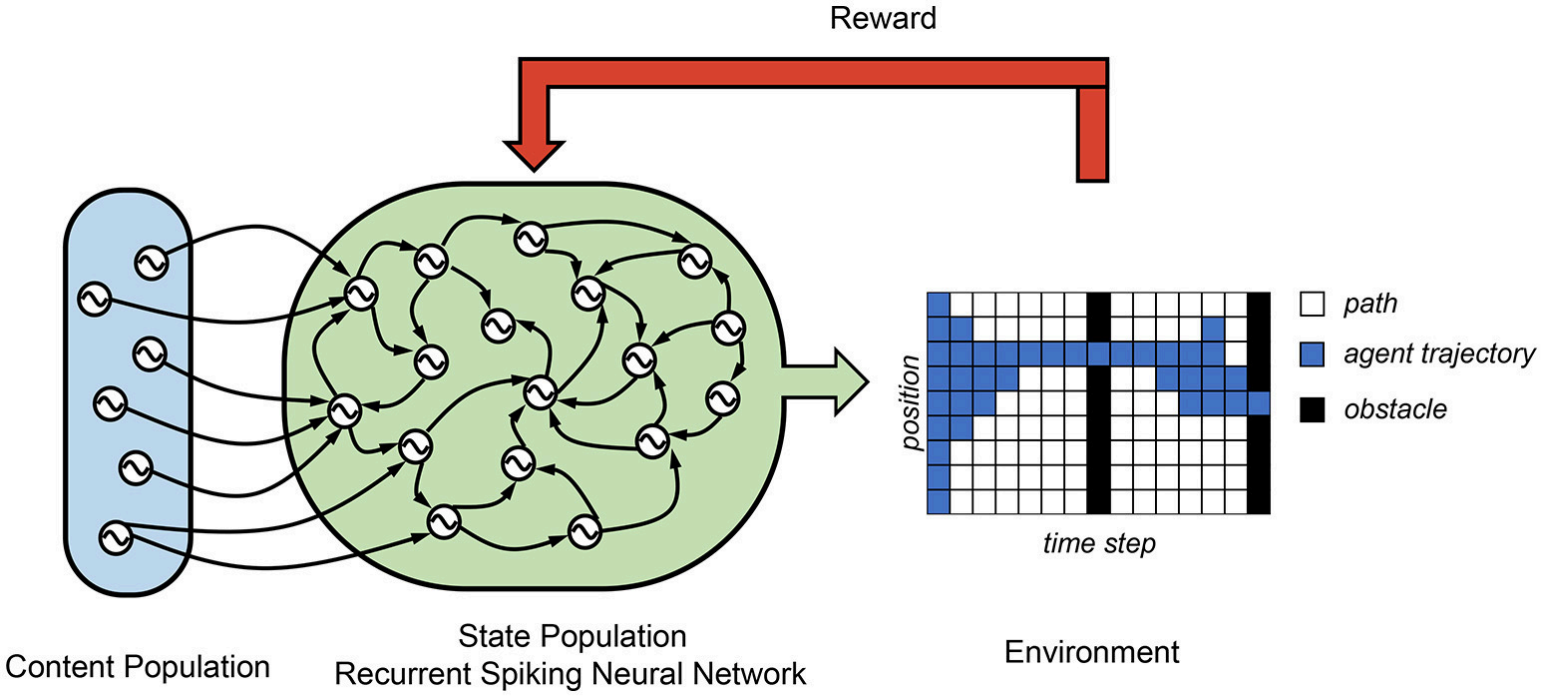
Control architecture of recurrent-SNN. A recurrent layer of state neurons is used to control the state of the agent and receives signals from the content population, which decides the target position according to different time step.

In Rueckert et al. (2016), a recurrent SNN is proposed for solving planning tasks, which consists of two populations of neurons, namely, the state neuron population and the content neuron population. (see Figure 5) The state neuron population consists of *K* state neurons, which control all the state of a freely moving target. In their finite horizon planning task, the agent spatial position is controlled by *nine* state neurons. These state neurons are wired to each other and the content neuron populations by R-STDP synapse. The context neurons produce spatiotemporal spike patterns that represent high-level goals and context information. In this case, its average firing rate represents the target spatial position at different time step. A final reward is only received if the agent passes through two obstacles, one at time *T*/2 and one at time *T*. They show that the optimal planning policy can be learned using the reward modulated update rule in a network where the state neurons follow winner-take-all (WTA) dynamics. Due to the probability, in each time step exactly one state neuron is active and encodes the current position of the agent. Their results demonstrated a successful planner trajectory planning task using a recurrent SNN.

## 5. Learning and robotics applications

Changes in the strength of synaptic connections between neurons are thought to be the physiological basis of learning (Vasilaki et al., 2009). These changes can either be gated by neuromodulators that encode the presence of reward or inner co-activation among neurons and synapses. In control tasks presented in this section, the network is supposed to learn a function that maps some state input to a control or action output. When successfully learned, the network is able to perform simple tasks such as wall following, obstacle avoidance, target reaching, lane following, taxi behavior, or food foraging. In most cases, the network input directly comes from the robot's sensors, which range from binary sensors, e.g., olfactory, to multi-dimensional continuous sensors, such as cameras. In other cases, the input can be pre-processed data, e.g., coming from electroencephalography (EEG) data. Similarly, the output can range from one-dimensional, binary behavior control to multi-dimensional continuous output values, e.g., for motor control, as well.

Initially, solving simulated control tasks was done by manually setting network weights, e.g., in Lewis et al. (2000) and Ambrosano et al. (2016). However, this approach is limited to solving simple behavioral tasks such as wall following (Wang et al., 2009) or lane following (Kaiser et al., 2016), it is usually only feasible for very small network architectures with few weights.

Therefore, a variety of training methods for SNNs in control tasks has been researched and published. Instead of focusing on criteria such as field of research, biological plausibility or the specific task, this section is meant to serve as a classification of published algorithms into the basic underlying training mechanisms from a robotics and machine learning perspective. In the first part of this section, some implementations of SNN control are introduced that use some form of Hebbian-based learning. In the second part, publications are shown that try to bridge the gap between classical reinforcement learning and spiking neural networks. Finally, some alternative methods on how to train and implement spiking neural networks are discussed.

### 5.1. Hebbian-based learning

One of the earliest theories in neuroscience explaining the adaption of synaptic efficacies in the brain during the learning process was introduced by Donald Hebb in his 1949 book *The Organization of Behavior* (Hebb, 1949). Often summarized by the phrase “*Cells that fire together, wire together,”* his idea is usually expressed in mathematical terms as

(8) $$\begin{array}{r} \Delta w_{ij}\propto v_{i}v_{j}, \end{array}$$

where *w*_*ij*_ refers to the change of synaptic weight between the presynaptic neuron *i* and the postsynaptic cell *j*; and *v* represents the activities of those neurons, respectively.

Hebbian-based learning rule that rely on the precise timing of pre and post-synaptic spikes play a crucial part in the emergence of highly non-linear functions in SNNs. Learning based on Hebbs rule has been successfully applied to problems such as input clustering, pattern recognition, source separation, dimensionality reduction, formation of associative memories, or formation of self-organizing maps (Hinton and Sejnowski, 1999). Furthermore, different biologically plausible learning rules have been used for using Spiking Neural Networks in robot control tasks. However, as the basic underlying mechanism stays the same, training these networks can be achieved in different ways as follows (see Table 1). In the table, the two-wheel vehicle means a vehicle with two active wheels.

**Table 1:** Learning rules based on STDP/Hebbian learning.

**Learning rule**	**Robot**	**Sensor**	**Methodology**	**Reference**
Unsupervised	Two-wheel vehicle	5 Proximity Sensors	Implementing an SNN on a resistive memory device and apply it to navigation tasks	Sarim et al., 2016a,b
	Mobile vehicle Casis-I	16 Ultrasonic Sensors	A behavior-based target-approaching navigation controller composed of three sub-controllers: the obstacle-avoidance, wall-following, and goal-approaching SNN controllers.	Wang et al., 2014, 2008
	TriBot Robot	Distance Sensor, Contact Sensor,	Using SNN to make robot navigate in an unknown environment and avoid obstacles	Arena et al., 2010
Supervised	Two-wheel insect	4 Proximity Sensors	Implementing an indirect training SNN in digital CMOS to navigate with obstacles	Hu et al., 2014; Mazumder et al., 2016
	Two-wheel insect	2 Terrain, 2 Target	Indirectly train an SNN by RBFs to determine precise spike timings and minimize a desired objective function	Zhang et al., 2013
	Aircraft	IMU	Indirectly training an SNN to approximate an optimal flight controller	Foderaro et al., 2010
	4-DoF robotic arm	4 Joint Encoder, 3 Spatial direction of end-effector	Using supervised learning to train a single-layer network to control a robotic arm with 4 degrees of freedom in 3D space	Bouganis and Shanahan, 2010
	2-DoF robotic arm	Sensorimotor	Using supervised learning to train a spiking model of the cerebellum to control a robotic arm	Carrillo et al., 2008
**STDP / Hebbian Learning**				
Conditioning	Simulated fly	Olfactory Receptor	Implementing an SNN inspired by Drosophila olfactory system to simulate flight	Faghihi et al., 2017
	Lego EV3 robotic platform	Camera, Infrared sensor Colour/light sensor	Learning and unlearning autonomously locomotion based on visual-input with reinforced/aversive reflex-response	Jimenez-Romero, 2017
	Two-wheel robot	3 Proximity Sensors, 1 RGB Sensor	Using Reward-dependent STDP learning rule to allow OC and CC learning	Dumesnil et al., 2016a,b
	Foraging Ants	Olfactory Sensors, Nociceptor	Learns to associate olfactory sensor input with different behaviors through a single-layer SNN	Jimenez-Romero et al., 2015, 2016
	Lego NXT 2.0	Color sensor, Touch Sensor	Using SNN to sustain OC in multiple learning scenarios	Cyr et al., 2014; Cyr and Thériault, 2015
	Two-wheel Vehicle	Light Sensors	Using light sensors in a target-reaching task to punish wrongful behavior	Iwadate et al., 2014
	Two-wheel Vehicle	5 Proximity Sensors, 9 IR Sensors, Vibration Sensor	Using infrared, ultrasound and visual neurons as CS and vibration neurons as US	Cyr and Boukadoum, 2012
	Mobile vehicle Casis-I	16 Ultrasonic Sensors	A learning algorithm combining operant conditioning and a shunting neural dynamics model is applied to the path planning	Wang et al., 2012
	TriBot Robot	Distance Sensor, Camera, Contact Sensor,	Using target distance as CS, while contact sensors work as US causing an unconditioned response	Arena et al., 2009a,b
R-STDP	Flapping Insect	GPS and IMU	Indirectly training an SNN-based controller for adaptive flight control	Clawson et al., 2016
	1-DoF robotics arm	5 Proximity Sensors	Using an SNN trained by a global reward and punishment signal to reach arbitrary targets	Spüler et al., 2015
	Musculoskeletal arm, WAM robot	Encoders	Using a cortical spiking model composed of several hundred spiking model-neurons to control a two-joint arm	Dura-Bernal et al., 2015
	CARL-SJR	Tactile Sensors	Using SNN to provide feedback to users by displaying bright colors on its surface.	Chou et al., 2015
	Two-wheel vehicle	2 Proximity Sensors	Implement a version of DA-modulated STDP on a food foraging task	Evans, 2015
	Foraging Simulator	Visual Sensors	Using reward-STDP based SNN to solve a grid-based foraging task	Skorheim et al., 2014
	DfRobotShop Rover	Camera, Light Sensor	Using an SNN and external flash to reinforce the goal-directed and adaptive behaviors	Helgadottir et al., 2013
	2-DoF robotics arm	Sensorimotor	Using an SNN based on R-STDP to control a two-joint virtual arm to reach to a fixed target	Neymotin et al., 2013
	1-DoF robotics arm	Encoder	Using an SNN to control a single-joint arm for target reaching	Chadderdon et al., 2012

#### 5.1.1. Unsupervised learning

According to STDP, if a presynaptic spike preceded a postsynaptic spike, a potentiation of the synaptic strength could be observed [Long Term Potentiation (LTP)], while the reversed order caused a depression [Long Term Depression (LTD)]. Because of the absence of direct goals, correction functions or a knowledgeable supervisor, this kind of learning is usually categorized as unsupervised learning (Hinton and Sejnowski, 1999). Learning based on STDP rule has been successfully applied to many problems such as input clustering, pattern recognition, and spatial navigation and mental exploration of the environment.

Wang et al. (2008) used this approach to train a behavior controller based on SNN to achieve obstacle avoidance using ultrasonic sensory signals with a mobile robot by driving it from different start positions. Compared with other classical NNs, they demonstrated that SNN needs fewer neurons and is relatively simple. Afterwards, they (Wang et al., 2014) extended their navigation controllers with with wall-following and goal-approaching abilities. In a similar research, Arena et al. (2010) presented an SNN based on an unsupervised learning paradigm to allow the robot to autonomously learn how to navigate in an unknown environment. Their controller allowed the robot to learn high-level sensor features, based on a set of basic reflexes, depending on some low-level sensor inputs by continuously strengthening the association between the unconditioned stimuli (contact and target sensors) and conditioned stimuli (distance and vision sensors).

#### 5.1.2. Supervised learning

In non-spiking neural networks, many successes in recent years can be summarized as finding ways to efficiently learn from labeled data. This type of learning, where a neural network mimics a known outcome from given data is called supervised learning (Hastie et al., 2001). A variety of different neuroscientific studies has shown that this type of learning can also be found in the human brain (Knudsen, 1994), e.g., in motor control and motor learning (Thach, 1996; Montgomery et al., 2002). But despite the extensive exploration of these topics, the exact mechanisms of supervised learning in biological neurons remain unknown.

**Figure 6:**
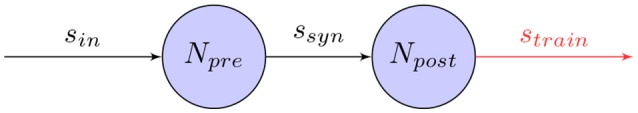
Supervised Hebbian training of a synapse: The weight of the synapse between pre and post-synaptic neurons, *N*_*pre*_ and *N*_*post*_, is adjusted by the timing of the pre-synaptic spike-train *s*_*syn*_ and external post-synaptic training signal *s*_*train*_.

Accordingly, a simple way of training SNNs for robot control tasks is by providing an external training signal that adjusts the synapses in a supervised learning setting. As shown in Figure 6, when an external signal is induced into the network as a post-synaptic spike-train, the synapses can adjust their weights, for example, using learning rules such as STDP. After an initial training phase, this will cause the network to mimic the training signal with satisfactory precision. Even though this approach provides a simple, straight-forward way for training networks, it is dependent on an external controller. Especially for control tasks involving high-dimensional network inputs, this may not be feasible.

Several models have been proposed on how this might work, either by using activity templates to be reproduced (Miall and Wolpert, 1996) or error signals to be minimized (Kawato and Gomi, 1992; Montgomery et al., 2002). In the nervous system, these teaching signals might be provided by sensory feedback or other supervisory neural structures (Carey et al., 2005). One of these models that is primarily suitable for single-layer networks is called supervised Hebbian learning (SHL). Based on the learing rule derived in 8, a teaching signal is used to train the postsynaptic neuron to fire at target times and to remain silent at other times. It can be expressed as

(9) $$\begin{array}{r} w_{ij}^{new}=w_{ij}^{old}+\alpha v_{i}t_{j}, \end{array}$$

where *w*_*ij*_ again is the synaptic efficacy between a presynaptic neuron *i* and a postsynaptic neuron *j*, α is the learning rate, *v*_*i*_ is the presynaptic neurons activity and *t*_*j*_ represents the postsynaptic teaching signal.

Carrillo et al. (2008) used this basic approach to train a spiking model of the cerebellum to control a robotic arm with 2 degrees of freedom in a target-reaching task taking joint angles and speeds, as well as target position as inputs. The spiking cerebellum model is trained by simulating the robotics arm to seven different targets repeatedly. In contrast to other STDP learning rules, only long-term depression was externally induced by a training signal, which relied on the motor error, namely the difference between the desired and actual state. In a similar experiment, Bouganis and Shanahan (2010) trained a single-layer network to control a robotic arm with 4 degrees of freedom in 3D space. As inputs, joint angles and the spatial During each training iteration, all four joints are driven with random motor commands, in the range of [−5°, 5°].direction of the end-effector were used, while outputs consisted of four motor-command neurons. The training signal was computed using an inverse kinematics model of the arm, adjusting the synaptic weights with a symmetric STDP learning rule. More examples can be found in Table 1 with an order by descending year.

#### 5.1.3. Classical conditioning

**Figure 7:**
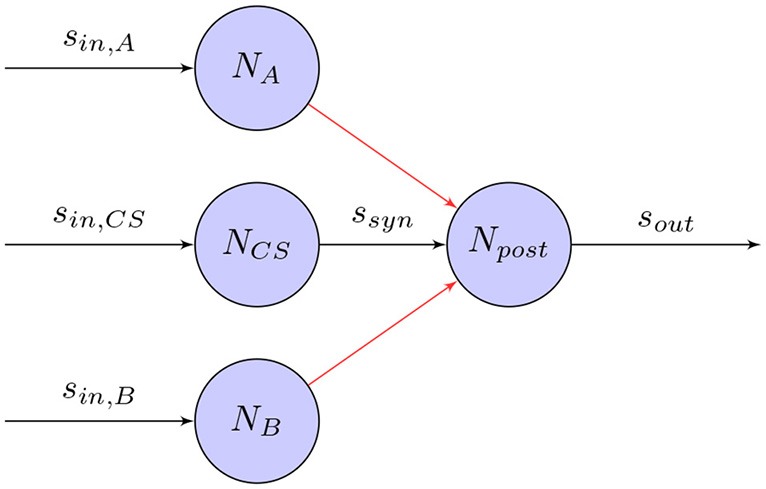
Classical Conditioning with STDP synapse between *N*_*pre*_ and *N*_*post*_: An unconditioned stimulus (US) A or B causes the post-synaptic neuron *N*_*post*_ to fire. The conditioned stimulus (CS) firing shortly before its associated US will adjust its weights so that *N*_*post*_ will fire even in the absence of US. Due to the Hebbian learning rule, the synaptic weight is unchanged when the other, unrelated stimulus causes *N*_*post*_ to fire.

Classical conditioning (Wikipedia, 2017a) refers to a learning procedure in which a biologically potent stimulus (e.g., food) is paired with a previously neutral stimulus (e.g., a bell). It will result that the neutral stimulus comes to elicit a response (e.g., salivation), which is usually elicited by the potent stimulus. In the famous experiment on classical conditioning (Pavlov and Anrep, 2003), Pavlov's dog learns to associate an unconditioned stimulus (US), in this case food, and a conditioned stimulus (CS), a bell, with each other. While, it is not clear how the high-level stimuli given in his experiment are processed within the brain, the same learning principle can be used for training on a neural level as well. Figure 7 shows how a synapse based on the STDP learning rule can associate US and CS provoking a response even in the absence of US.

Following this principle, bio-inspired robots can learn to associate a CS, e.g., sensory information, with a US that functions as an external reinforcer. That way, robots can learn to follow the desired behavior based on sensory inputs. Arena et al. (2009a,b, 2010) showed how classical conditioning can be used in an obstacle avoidance and target reaching task. In an SNN with two output motor neurons, distance and vision sensors function as CS, while contact and target sensors work as US causing an unconditioned response. By navigating the robot in a pre-designed enclosed environment, the robot successfully learned to associate the CS and the US together and reach the target without hitting obstacles. In a similar experiment, Cyr and Boukadoum (2012) carried out different classical conditioning tasks in a controlled virtual environment using infrared, ultrasound and visual neurons as CS and vibration neurons as US. Wang et al. (2008, 2014) constructed a controller that stimulated two motor neurons as US. A single-layer SNN using proximity sensor data as CS input was then trained in tasks such as obstacle avoidance and target reaching. Iwadate et al. (2014) used light sensors in a target-reaching task to punish wrongful behavior. Jimenez-Romero et al. (2015, 2016) and Jimenez-Romero (2017), implemented a virtual ant that learns to associate olfactory sensor input with different behaviors through a single-layer SNN. The robot was able to learn to recognize rewarding and harmful stimuli as well as simple navigation in a simulated environment. Casellato et al. (2014) proposed a realistic cerebellar SNN with a real haptic robotic arm to achieve diverse sensorimotor tasks. In all tasks, the robot learned to adjust timing and gain of the motor response and successfully reproduced human biological systems acquire, extinguish, and express knowledge in a noisy world.

In order to successfully learn such behavioral tasks, some unconditioned stimulus has to be given for every relevant conditioned stimulus that the robot should learn. This also means that the robot will learn to associate stimuli that are delayed in time. Taken together, using classical conditioning for robot control basically means constructing an external controller that provides unconditioned stimuli for every relevant state input, which may not be feasible in many tasks.

#### 5.1.4. Operant conditioning

While classical conditioning is concerned with passively associating conditioned and unconditioned stimuli with each other, operant conditioning (OC) consists of associating stimuli with responses and actively changing behaviors thereafter. Conceptually, operant conditioning involves changing voluntary behaviors and is closely related to reinforcement learning and its agent-environment interaction cycle. A behavior response is followed by either reinforcement or punishment. Reinforcement following a behavior will cause the behavior to increase, but if behavior is followed by punishment the behavior will decrease. Instead of developing a formal mathematical model, operant conditioning has been mainly researched in biological and psychological domains. Despite advances in the understanding of operant conditioning, it is still not clear how this type of learning is implemented on a neural level.

In this context, Cyr et al. (2014) and Cyr and Thériault (2015) developed a spiking OC model that consists of an input feeding cue neuron, an action neuron and a predictor neuron that receives rewards or punishments. With this simple basic architecture and learning rules such as habituation and STDP, they were able solve simple OC-related tasks in a simulated environment, such as pushing blocks. In another publication by Dumesnil et al. (2016a,b) a reward-dependent STDP learning rule was implemented on a robot to allow for OC learning and demonstrated in a maze task. The RGB camera was used to capture the color information which represented the cue or the reward in the maze environment. Eventually, the robot learned the association, if an action was frequently followed by a reward.

#### 5.1.5. Reward-modulated training

**Figure 8:**
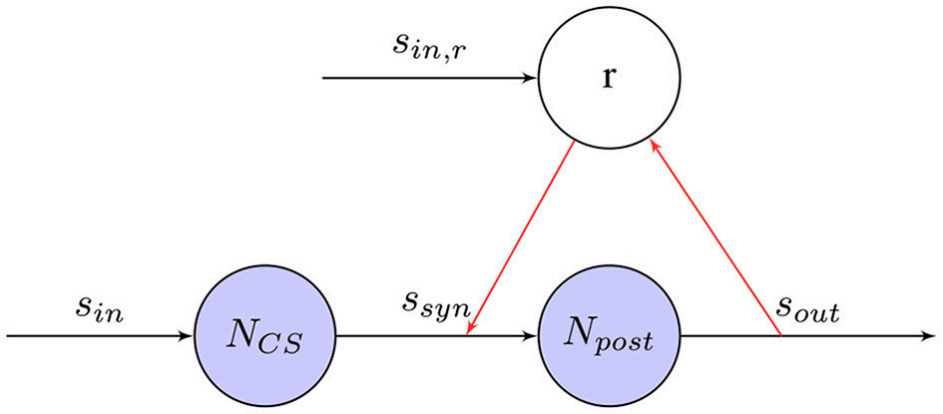
Reward-modulated STDP synapse between *N*_*pre*_ and *N*_*post*_: Depending on the post-synaptic output spike-train, a reward *r* is defined that modulates the weight change of the synapse.

In Figure 8 the learning rule for reward-based training is shown. Using one or more chemicals emitted by a given neuron to regulate diverse populations of neurons is know as neuromodulation (Hasselmo, 1999). As one of the neuromodulators, dopamine neurons forming the midbrain dopaminergic cell groups are crucial for executive functions, motor control, motivation, reinforcement, and rewards. Most types of neurological rewards increase the level of dopamine in the brain, thus stimulating the dopamine neurons (Schultz, 1998). Inspired by dopaminergic neurons in the brain, the effects of STDP events are collected in an eligibility trace and a global reward signal induces synaptic weight changes. In contrast to supervised training as discussed before, rewards can be attributed to stimuli, even if they are delayed in time. This can be a very useful property for robot control, because it might simplify the requirements of an external training signal leading to more complex tasks. A simple learning rule combing models of STDP and a global reward signal was proposed by Florian (2007) and Izhikevich (2007). In the R-STDP, the synaptic weight *w* changes with the reward signal *R*. The eligibility trace of a synapse can be defined as,

(10) $$\begin{array}{r} ċ\left(t\right)=-\frac{c}{\tau_{c}}+\omega \left(\Delta t\right)\delta \left(t-s_{pre/post}\right)C_{1} \end{array}$$

where *c* is an eligibility trace. *s*_*pre*/*post*_ means the time of a pre- or post-synaptic spikes. *C*_1_ is a constant coefficient. τ_*c*_ is a time constant of the eligibility trace. δ(·) is the Dirac delta function.

(11) $$\begin{array}{r} ẇ\left(t\right)=R\left(t\right)\times c\left(t\right) \end{array}$$

where *R*(*t*) is the reward signal.

In the literature, a variety of algorithms has been published using this basic learning architecture for training. Even though they are all based on the same mechanism, the rewards can be constructed in different ways.

1. Rewarding Specific Events: The most straight-forward implementation of reward-based learning resembling classical reinforcement learning tasks uses rewards associated to specific events. Evans (2012) trained a simple, single-layer SNN in several food foraging tasks consisting of 4 input sensor neurons and 4 output motor neurons. In a separate network, other reward-related sensor neurons stimulated a dopaminergic neuron that in turn modulated the synaptic weight change. With this simulation setup, the robot was able to learn food-attraction behavior and subsequently unlearn this behavior when the environment changed. This was achieved by a training stage during which the robot were randomly droved to explore the environment effectively. By shifting the dopamine response from the primary to a secondary stimulus, the robot was able to learn, even with large temporal distance, between correct behavior and reward. Faghihi et al. (2017) showed an SNN model of a fruit fly that is able to execute both first and second order conditioning. In a simple task, the simulated fly learned to avoid getting close to an olfactory target emitting electric shocks. Furthermore, the same behavior can be transferred to a secondary stimulus that is associated to the primary stimulus without emitting electric shocks itself.
2. Control Error Minimization: As opposed to rewarding specific events, dopamine-modulated learning can also be used in an optimization task to minimize an objective function. This is usually achieved by strengthening or weakening the connections that lead to changes in the objective function based on their eligibility traces. Clawson et al. (2016) used this basic architecture to train an SNN to follow a trajectory. The network consisted of lateral state variables as inputs, a hidden layer and an output layer population decoding the lateral control output. Learning is achieved offline by minimizing the error between decoded actual and desired output, which is provided by an external linear controller.
3. Indirect Control Error Minimization: For some potential applications of SNNs, e.g., neuroprosthetic devices implanted in the brain, direct manipulation of synaptic weights might not be possible. Therefore, an indirect approach to training SNNs was shown by Foderaro et al. (2010) that induces changes in the synaptic efficacy through input spikes generated by a separate critic SNN. This external network was provided with control input as well as feedback signals and trained using a reward-based STDP learning rule. By minimizing the error between control output and optimal control law offline, it was able to learn adaptive control of an aircraft. Similar ideas were presented by Zhang et al. (2012), Zhang et al. (2013), Hu et al. (2014), and Mazumder et al. (2016) who trained a simple, virtual insect in a target reaching and obstacle avoidance task.
4. Metric Minimization: The same principle can also be applied to minimize a global metric that might be easier to construct and calculate than an external controller. Chadderdon et al. (2012) proposed a spiking-neuron model of the motor cortex which controlled a single-joint arm in a target-reaching task. The model consisted of 144 excitatory and 64 inhibitory neurons with proprioceptive inputs cells and output cells controlling the flexor and extensor muscles. A global reward or punishment signal was given depending on the change of hand-target distance during the learning phase, during which the robot was set with five different targets repeatedly. Neymotin et al. (2013) and Spüler et al. (2015) extended this architecture with two-joint robotic arm later. Similarly, Dura-Bernal et al. (2015) used a biomimetic cortical spiking model composed of several hundred spiking model-neurons to control a two-joint arm. With proprioceptive sensory input (muscle lengths) and muscle excitation output, the network was trained by minimizing the hand-target distance. The distance error was reduced by repeatedly move to the target with the guidance of a global reward/punish signal. Kocaturk et al. (2015) extended the same basic architecture in order to develop a brain-machine interface. Extracellularly recorded motor cortical neurons provide the network inputs used for prosthetic control. By pressing a button, the user can reward desired movements and guide the prosthetic arm toward a target. Using a miniaturized microprocessor with resistive crossbar memories implemented on a two-wheeled differential drive robot, Sarim et al. (2016a,b) showed how an STDP-based learning rule could lead to target approaching and obstacle avoidance behavior. Although, in this case, learning was implemented using if-then rules that relied on distance changes from target and obstacles, it is conceptually identical to reward-modulated learning. This can easily be seen by exchanging the if-rules with a reward of +1 or −1.
5. Reinforcing Associations: Chou et al. (2015) introduced a tactile robot that uses a network architecture inspired by the insular cortex. As in classical conditioning, a dopamine-modulated synaptic plasticity rule was used to reinforce associations between conditioned and unconditioned stimuli.

### 5.2. Reinforcement learning

In the previous subsection, a variety of approaches was presented for training SNNs based on Hebbian learning rules. This was done either by providing a supervised training signal through an external controller or by using a reward-based learning rule with different ways of constructing the reward. The latter type of learning, however, was shown to successfully train SNNs in simple tasks solely based on delayed rewards. In general, all of these approaches have been trained in tasks that don't require looking very far ahead, as reinforcement learning theories usually do.

In classical reinforcement learning theory, on the other hand, learning to look at multiple steps in advance in a Markov Decision Process (MDP) is one of the main concerns. Therefore, several algorithms have been published combining SNNs with classical reinforcement learning algorithms.

#### 5.2.1. Temporal difference

The learning rule in which one looks at one or more steps forward in time was introduced as temporal difference (TD) learning. Hereby, Potjans et al. (2009) and Frémaux et al. (2013) used place cells to represent the state space in an MDP and single-layer SNNs for state evaluations and policies. Both algorithms were able to learn to navigate in a simple grid-world after some training. With a similar approach, Nichols et al. (2013) presented a robot controller inspired by the control structures of biological systems. In a self-organizing, multi-layered network structure, sensory data coming from distance and orientation sensors was gradually fused into state neurons representing distinct combinations of sensory inputs. On top, each individual state neuron was connected to 3 output motor neurons. By fusing the sensory input into distinct state neurons and connecting them to action neurons, a simplified TD learning rule could be used to set each synaptic weight in the last layer individually, when the robot conducted a trial locomotion. Performance of this controller was demonstrated in a wall-following task.

While these state representations work very well for relatively small state spaces, they are usually bound to fail for larger, high-dimensional state spaces, since the TD method can only obtain the reward in several steps. Therefore, it is less stable and may converge to the wrong solution, especially for high-dimensional state spaces. In fact, these approaches can conceptually be seen as an SNN implementation of table-based Q-learning.

#### 5.2.2. Model-based

Although for robot control tasks, such as those shown in this paper, model-free reinforcement learning methods seem favorable, two recent publications are at least worth mentioning that presented SNN implementations of model-based reinforcement learning algorithms. Rueckert et al. (2016) presented a recurrent spiking neural network for planning tasks that was demonstrated on a real robot in an obstacle avoidance task. Friedrich and Lengyel (2016) implemented a biologically realistic network of spiking neurons for decision making. The network uses local plasticity rules to solve one-step as well as sequential decision making tasks, which mimics the neural responses recorded in frontal cortices during the execution of such similar tasks. Their model reproduced behavioral and neuro-physiological data on tasks ranging from simple binary choice to multi-step sequential decision making. They took a two-step maze navigation task as an illustration. During each state, the rat was rewarded with different values according to its actions. The reward was modeled as an external stimuli. The SNN learned a stable policy within 10 ms.

### 5.3. Others

Except for the two aforementioned major methods, there are also other training methods for SNNs in robot control tasks as follows (see Table 2).

**Table 2:** Other learning rules.

**Learning rule**		**Robot**	**Sensor**	**Methodology**	**Reference**
Evolutionary algorithms		*Neural Racing* game	Speedometer, Proximity Sensors	Using evolutionary algorithm to train SNN and compare results with multi-layer perceptron	Markowska and Koldowski, 2015
		Quadrotor	GPS	Using evolutionary algorithm to generate high utility topology/weight combinations in the SNN	Howard and Elfes, 2014
		Two-wheel Vehicle	5 IR Sensors	Using SNN to mimic the behaviors captured under control of a heuristic rule program	Batllori et al., 2011
		Khepera Robot (Two-wheel Vehicle)	Linear Camera	Using evolution to rapidly generate SNN capable of navigating in a textual environment	Floreano et al., 2006; Floreano and Mattiussi, 2001
		Two-wheel Vehicle	4 IR Sensors	A use-dependent synaptic modification algorithm of SNN for obstacle-avoidance vehicle behavior	Alnajjar and Murase, 2006
		Two-wheel Vehicle	9 Ultrasonic Sensors, 4 Bump Sensors	Using an adaptive GA to evolve the SNN online through interaction with the real environment	Hagras et al., 2004
Fuzzy logical		Two-wheel Vehicle	7 Ultrasonic Sensors (5 in front, 2 at back)	Using SNN to mimic the knowledge of a fuzzy controller	Kubota, 2004
Liquid state machine		Hexapod Robot	Visual Sensor (Distance, Height)	Mushroom bodies in drosophila are modeled as a recurrent SNN under LSM paradigm	Arena et al., 2017
		2-Dof Ball Balance Platform	Position and Velocity	Using a cortical network (LSM) to learn under a supervised learning rule for position control	Probst et al., 2012
		Khepera Robot	8 IR Sensors	Using Randomly generated recurrent SNN to operate real-time obstacle avoidance	Burgsteiner, 2005

#### 5.3.1. Evolutionary algorithms

In nature, evolution has produced a multitude of organisms in all kinds of shapes with survival strategies optimally aligned to environmental conditions. Based on these ideas, a class of algorithms has been developed for finding problem solutions by mimicking elementary natural processes called evolutionary algorithms (Michalewicz, 1996). Generally, evolutionary processes can be understood as some form of gradient-descent optimization. Therefore, a typical problem using these algorithms is getting stuck in local minima. In applications in robot control, evolving SNNs have been shown to work well in mostly static environments. Due to the training principle of trial and error, there are usually difficulties in dynamically changing environments.

Floreano and Mattiussi (2001) showed a vision-based controller in an irregularly textured environment that navigated without hitting obstacles. The predefined SNN consisted of 18 sensory-input receptors connected to 10 fully-connected hidden neurons and 2 motor-output neurons. Using static synaptic weight values, the algorithm was used to search the space of connectivity by genetically evolving only signs of weights (excitatory and inhibitory), when the robot was continuously driving around in the experiment setup. With a population of 60 individuals, fitness was evaluated by summing up over motor speeds at every time step, and new generations were created using one-point crossover, bit mutation and elitism. Hagras et al. (2004) later extended this approach to evolving SNN weights as well using adaptive crossover and mutation probabilities. They were able to evolve good SNN controllers in a small number of generations in a wall-following scenario. Howard and Elfes (2014) presented a quadrotor neurocontroller that performed a hovering task in challenging wind conditions. With a feed-forward network taking the differences between current position and target position as input and pitch, roll and thrust as output, weights and topology were evolved to minimize the spatial error. In a target-reaching and obstacle-avoidance task using binocular light sensors and proximity sensors, Batllori et al. (2011) evolved an SNN by minimizing the control error in order to mimic an external controller signal. Markowska and Koldowski (2015) used a feed-forward network architecture of predefined size to control a toy car. Based on speed, localization and road boarder input signals, the network controlled speed regulation and turn direction, and evolved its weights using a genetic algorithm.

#### 5.3.2. Self-organizing algorithms

Alnajjar and Murase (2006) formulated a synaptic learning rule that enforced connections between neurons depending on their activities. During the learning phase, the robot gradually organized the network and the obstacle avoidance behavior was formed. With this self-organization algorithm that resembles other Hebbian-based learning methods, they were able to learn obstacle avoidance and simple navigation behavior.

#### 5.3.3. Liquid state machine

As a particular kind of SNN, an liquid state machine (LSM) usually consists of a large assemblage of neurons that receives time-varying input from external sources as well as from other neural units (Yamazaki and Tanaka, 2007). All mixed and disorderly neuron units are randomly generated and then arranged under the activations of recurrent spatio-temporal patterns of the connections obtained from the time-varying input. Hence, the LSM is regarded as a large variety of nonlinear functions which is able to compute the output as linear combinations of the input. LSMs seem to be a potential and promising theory to explain brain operation mainly because neuron activities are not hard coded and limited for specific tasks. Burgsteiner (2005), Probst et al. (2012), and Arena et al. (2017) showed how liquid state machines can be trained for robot control tasks.

## 6. Simulators and platforms

With the fast development of neuroscience and chip industry, large-scale neuromorphic hardware using spiking neural networks has been studied to achieve the same capabilities as animal brains in terms of speed, efficiency, and mechanism. For examples, SpiNNaker (Furber et al., 2013) is a million-core system for modeling large-scale SNNs in real time. TruthNorth (Merolla et al., 2014) contains 1 million programmable spiking neurons and only consumes less than one hundred milliwatts. Other neuromorphic computing platforms such as Neural Grid (Benjamin et al., 2014), NeuroFlow (Cheung et al., 2016) can be found and introduced in Schuller and Stevens (2015). Meanwhile, a growing number of dynamic simulators has been developed to assist robotic research (Ivaldi et al., 2014), such as Gazebo (Koenig and Howard, 2004), ODE (Wikipedia, 2017c), and V-Rep (Rohmer et al., 2013). Those simulators greatly facilitate the research process that involving mechanical design, virtual sensors simulation, and control architecture.

Although adequate tools exist to simulate either spiking neural networks (Brette et al., 2007; Bekolay et al., 2014), or robots and their environments (Staranowicz and Mariottini, 2011; Harris and Conrad, 2011), tools that offer researchers joint interaction, including a realistic brain model, robot, and sensory-rich environment, are in need. Some existing platforms are listed in Table 3.

**Table 3:** Taxonomy of platforms for robotics control based on SNN.

**Platform**		**Name**	**Methodology**	**Reference**
Platform		Neurorobotics Platform	Design, import, and simulate different robot bodies and diverse brain models in rich environments	Falotico et al., 2017
		Musculoskeletal Robots	Combining Myorobotics with SpiNNaker the proof of principle of a system that can scale to dozens of neurally controlled, physically compliant joints.	Richter et al., 2016
		Retina simulation	The retina simulation platform is integrated in the NRP.	Ambrosano et al., 2016
		Neural self-driving vehicle simulation framework	A visual encoder from camera images to spikes inspired by the silicon retina, and a steering-wheel decoder based on an agonist antagonist muscle model.	Kaiser et al., 2016
		iSpike	Interface between SNN simulators and the iCub humanoid robot	Gamez et al., 2012
		AnimatLab	Provide functions, such as robot modeling, two neural models, and plugins for importing other models.	Cofer et al., 2010

iSpike (Gamez et al., 2012), as the first attempt to combine spiking neural networks and robots, is a C++ library that provides an interface between SNN simulators and the iCub humanoid robot. It uses a biologically inspired approach to convert the robot's sensory information into spikes that are passed to the neural network simulator, and it decodes output spikes from the network into motor signals that are sent to control the robot. CLONES (Voegtlin, 2011) communicates between the Brian neural simulator (Goodman and Brette, 2009) and SOFA (Allard et al., 2007) and is also an interface used for shared memory and semaphores. A more generic system which permits dealing with simulated robotic platforms is AnimatLab (Cofer et al., 2010), which provides functionalities such as robot modeling, two neural models, and plugins for importing other models.

Recently, the first release of the HBP Neurorobotics Platform (NRP) (American Association for the Advancement of Science, 2016; Falotico et al., 2017) was presented, which was developed within the EU Flagship Human Brain Project. For the first time, it provides scientists with an integrated toolchain to connect pre-defined and customized brain models to detailed simulations of robot bodies and environments in *in-silico* experiments. In particular, NRP consists of six key components, which are essential to construct neurorobotics experiments from scratch. It can be seen that the NRP provides a complete framework for the coupled simulation of robots and brain models. The Brain Simulator simulates the brain by bio-inspired learning algorithms such as a spiking neural network to control the robot in a silico neurorobotics experiment. The World Simulator simulates the robots and their interacting environment. The Brain Interface and Body Integrator (BIBI) builds a communication channel between brain models and robot models. The Closed Loop Engine (CLE) is responsible for the control logic of experiments as well as for the data communication between different components. The *Backend* receives requests from the frontend for the neurorobotics experiment and distributes them to the corresponding component, mainly via ROS. The *Frontend* is a web-based user interface for neurorobotics experiments. Users are able to design a new experiment or edit existing template experiments.

## 7. Open research topics

In the previous sections, the state-of-the-art of SNN-based control for various robots has been surveyed in terms of learning methods. Although an increasing amount of work has been done to explore the theoretical foundations and practical implementations of SNNs for robotics control, many related topics need to be investigated, especially in the following areas.

### 7.1. Biological mechanism

Despite the extensive exploration of the functions and structure of the brain, the exact mechanisms of learning in biological neurons remain unknown. Some of those related to robotics applications are listed as: (1) How is diverse information coded in many neural activities other than the rates and timing of spikes? (2) How are memories distinguished, stored, and retrieved in such an efficient and precise manner? (3) How do brains simulate the future, since it involves the concept of “previous steps,” thus requiring some form of memory? As long as we can constantly address these unsolved mysteries of the brain, the robots of the future definitely can achieve more advanced intelligence.

### 7.2. Designing and training SNNs

None of the currently suggested algorithms are general-purpose able, at least in principle, to learn an arbitrary task in the way that backpropagation (through time) and its variants (with all their limitations) do for rate neurons (Grüning and Bohte, 2014). Therefore, there is no general design framework that could offer the functionalities of modeling and training, as well as those substantial tools for the conventional ANNs do, for instance, Tensorflow (Allaire et al., 2016), Theano (Theano Development Team, 2016), and Torch (Collobert et al., 2011). The nature of this situation is that training these kind of networks is notoriously difficult, especially when it comes to deep-network architectures. Since error backpropagation mechanisms commonly used in ANNs cannot be directly transferred to SNNs due to non-differentiabilities at spike times, there has been a void of practical learning methods.

Moreover, training should strengthen the combination with the burgeoning technologies of reinforcement learning, for instance, extending SNN into deep architecture or generating continuous action space (Lillicrap et al., 2015). In the future, combining the R-STDP with a reward-prediction model could lead to an algorithm that is actually capable of solving sequential decision tasks such as MDPs as well.

### 7.3. High performance computing with neuromorphic devices

Another important general issue that needs extensive research and is not clearly defined is how to integrate SNN-based controllers into neuromorphic devices, since they have the potential to offer fundamental improvements in computational capabilities such as speed and lower power consumption (Hang et al., 2003; Schuller and Stevens, 2015). These are of vital importance for robot applications, especially in mobile applications where real-time responses are important and energy supply is limited. An overview of how to program SNNs based on neuromorphic chips can be found (Walter et al., 2015b).

SNNs computation can highly benefit from parallel computing, substantially more so than conventional ANNs. Unlike a traditional neuron in rate coding, a spiking neuron does not need to receive weight values from each presynaptic neuron at each compution step. Since at each time step only a few neurons are active in an SNN, the classic bottleneck of message passing is removed. Moreover, computing the updated state of membrane potential is more complex than computing a weighted sum. Therefore communication time and computation cost are much more well-balanced in SNN parallel implementation as compared to conventional ANNs.

### 7.4. Interdisciplinary research of neuroscience and robotics

Another barrier that needs to be removed comes from a dilemma for the researchers of neuroscience and robotics: Roboticists often use a simplified brain model in a virtual robot to make a real-time simulation, while neuro-scientists develop detailed brain models that are not possible to be embedded into the real world due to their high complexity. Learning complex sensorimotor mapping of the robot generated in the interaction with dynamic and rich sensory environment is also required (Hwu et al., 2017). An ongoing solution is the Neurorobotics Platform, which offers adequate tools to model virtual robots, high-fidelity environments, and complex neural network models for both neuroscientists and roboticists.

## 8. Conclusion

By mimicking the underlying mechanisms of the brain much more realistically, spiking neural networks have showed great potential for achieving advanced robotic intelligence in terms of speed, energy efficiency, and computation capabilities. Therefore in this article, we seek to offer readers a comprehensive review of the literature about solving robotic control tasks based on SNNs as well as the related modeling and training approaches, and meanwhile offer inspiration to researchers. Specifically, we retrospect the biological evidences of SNNs and their major impetuses for being adopted for the area of robotics at the beginning. Then, we present the mainstream modeling approaches for designing SNNs in terms of neuron, synapse, and network. The learning solutions of SNNs are generally classified into two types based on Hebbian rule and reinforcement learning, illustrated and expounded with exhaustive robotic-related examples and summary tables. Finally, some popular interfaces or platforms for simulating SNNs for robotics are preliminarily investigated.

As indicated in the open topics, the biggest challenge for control tasks based on SNNs is a lack of a universal training method, as back-propagation is to the conventional ANNs. Therefore, more knowledge and interactions from the fields of neuroscience and robotics are needed to explore this area in the future.

## Author contributions

ZB, FR, and AK brought up the core concept and architecture of this manuscript. ZB, CM, FR, and KH wrote the paper.

### Conflict of interest statement

The authors declare that the research was conducted in the absence of any commercial or financial relationships that could be construed as a potential conflict of interest.
